# Regulation of lifespan by chemosensory and thermosensory systems: findings in invertebrates and their implications in mammalian aging

**DOI:** 10.3389/fgene.2012.00218

**Published:** 2012-10-18

**Authors:** Dae-Eun Jeong, Murat Artan, Keunhee Seo, Seung-Jae Lee

**Affiliations:** ^1^Division of Molecular and Life Science, Pohang University of Science and TechnologyPohang, South Korea; ^2^Information Technology Convergence Engineering, Pohang University of Science and TechnologyPohang, South Korea; ^3^School of Interdisciplinary Bioscience and Bioengineering, Pohang University of Science and TechnologyPohang, South Korea

**Keywords:** aging, sensory neuron, chemosensation, thermosensation, *Caenorhabditis elegans*, *Drosophila melanogaster*, endocrine signaling, hibernation

## Abstract

Many environmental factors that dynamically change in nature influence various aspects of animal physiology. Animals are equipped with sensory neuronal systems that help them properly sense and respond to environmental factors. Several studies have shown that chemosensory and thermosensory neurons affect the lifespan of invertebrate model animals, including *Caenorhabditis elegans* and *Drosophila melanogaster*. Although the mechanisms by which these sensory systems modulate lifespan are incompletely understood, hormonal signaling pathways have been implicated in sensory system-mediated lifespan regulation. In this review, we describe findings regarding how sensory nervous system components elicit physiological changes to regulate lifespan in invertebrate models, and discuss their implications in mammalian aging.

## Introduction

Organisms are subject to continuously changing environments and numerous stimuli that originate from various sources. These stimuli are perceived and processed by different mechanisms to allow the organism to respond appropriately. In most animals, these ambient cues are perceived by a system that consists of many sensory neurons and protects animals from harmful stimuli, such as burns and noxious poisons, by allowing them to avoid these damaging conditions. Therefore, the sensory system is of vital importance for many organisms to thrive in their niches.

**Figure 1:**
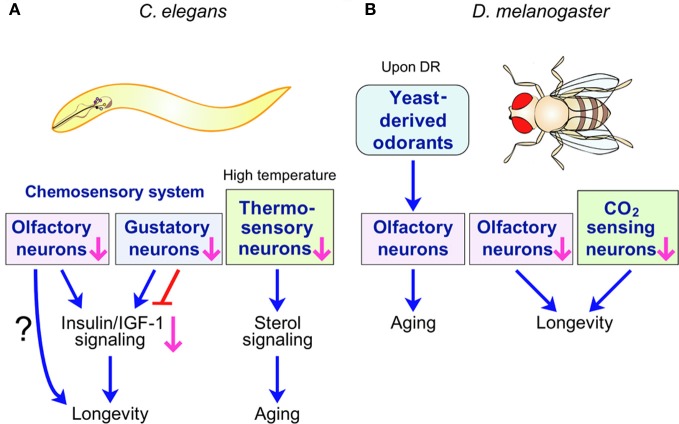
***C. elegans* and *D. melanogaster* sensory systems influence lifespan. (A)** *C. elegans* chemosensory and thermosensory systems regulate lifespan through hormonal signaling. Specific chemosensory (olfactory or gustatory) neurons promote or limit longevity. Insulin/IGF-1 signaling can mediate this longevity response downstream of chemosensory neurons. Perturbation of thermosensory system accelerates aging at high temperatures by influencing the sterol hormonal signaling pathway. **(B)** Chemosensory systems regulate *D. melanogaster* lifespan. Olfaction of nutrient-derived odorants promotes the aging of long-lived *D. melanogaster* upon dietary restriction (DR). Perturbation of the *D. melanogaster* olfactory system and inhibition of the CO_2_-sensing system both prolong lifespan. However, the signaling pathways regulated by *D. melanogaster* chemosensory systems to influence lifespan are unknown.

The perception of external stimuli and subsequent signal transmission for proper responses are the primary function of sensory neurons. These neurons were assigned another important function after remarkable discoveries in invertebrate model animals that lifespan is actively regulated by sensory neuronal systems. Inhibiting chemosensation modulates lifespan in *Caenorhabditis elegans* and *Drosophila melanogaster*, and defects in thermosensation shorten *C. elegans* lifespan at high temperature (Figure 1). Here, we describe how sensory neuronal systems regulate aging processes in these invertebrate model animals and speculate the implications of these findings with regard to mammalian aging.

## The role of the chemosensory system in aging

### Chemosensory systems of *C. elegans* and *D. melanogaster*

**Figure 2:**
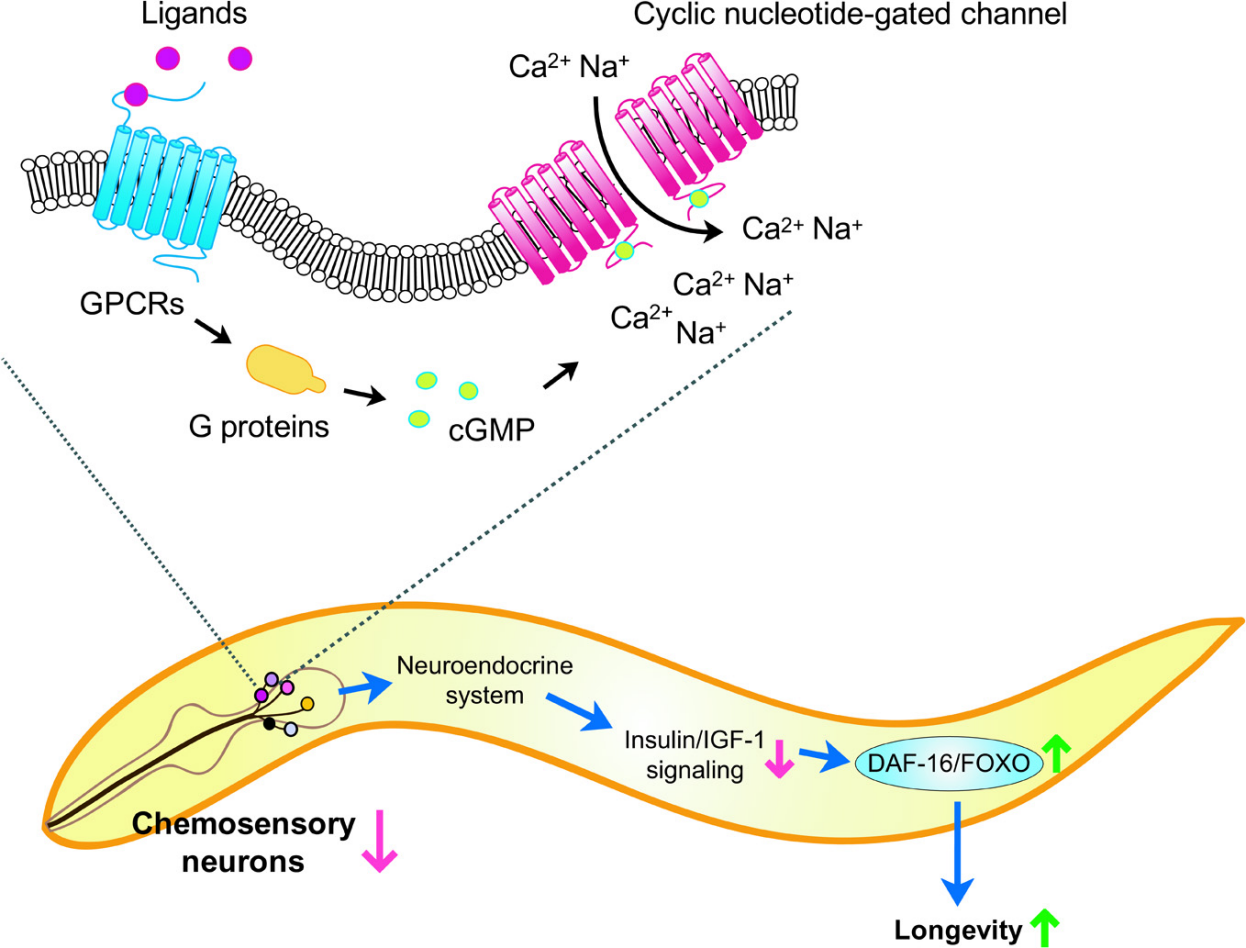
**Model of lifespan control by chemosensation and insulin/IGF-1 signaling in *C. elegans*.** A subset of ciliated neurons in the head region (for example, in the amphid) perceive chemosensory cues and trigger signal transduction cascades. Upon binding to ligands, chemosensory G protein-coupled receptors (GPCRs) activate G proteins, which lead to cGMP production. This in turn opens cyclic nucleotide-gated channels and increases Ca^2+^ and Na^+^ influx. Inhibiting chemosensory neuronal structure or function in specific neurons may alter neuroendocrine signaling and reduces insulin/IGF-1 signaling, which activates the DAF-16/FOXO transcription factor and promotes longevity.

Chemosensation is initiated by the detection of chemical cues by receptors in chemosensory neurons that form neural circuits with other neurons (Bargmann, 2006). Chemosensory neural circuits include motor neurons; therefore, the organism's sensory system can elicit behavioral responses to environmental stimuli. Model invertebrates with relatively simple nervous systems have been utilized to genetically dissect functions and mechanisms of chemosensory systems. One of them is the nematode *C. elegans*, a simple multi-cellular animal that lives in decomposing organic material. Approximately 60 of the 302 *C. elegans* neurons are ciliated sensory neurons, including chemosensory neurons, some of which are in the amphid organ in the head (Bargmann, 2006) (Figure 2). Chemosensory signals are transduced by many effector proteins in the neurons, including G protein-coupled receptors (GPCRs) that are activated by binding with their ligands (Figure 2). GPCRs activate G protein signaling to influence the level of cyclic GMP (cGMP), which functions as a second messenger for the chemosensory signal transduction. cGMP binds to and opens cyclic nucleotide-gated channels to regulate cation flux that is required for chemosensation (Bargmann, 2006).

Another invaluable invertebrate model organism that has been used to understand the chemosensory neural system is the fruit fly *D. melanogaster*. The fly genome expresses 62 odorant receptors in approximately 2500 olfactory receptor neurons (ORNs), which are covered by sensilla. These structures cover the third segment of antenna and maxillary palp, which are specialized olfactory appendages in the head that sense environmental odors (Vosshall and Stocker, 2007). Olfactory neurons innervate glomeruli in the antennal lobe and target to local interneurons that connect with cholinergic projection neurons to form neural circuits for signal transmission. In contrast to the olfactory neurons, gustatory neurons are gathered in taste bristles on the body of *D. melanogaster*, including the proboscis, legs, and wings. This wide distribution of gustatory organs may allow *D. melanogaster* to seek preferred foods and find appropriate egg-laying locations (Vosshall and Stocker, 2007).

### Chemosensory perturbation influences the lifespan of *C. elegans* and *D. melanogaster*

Studies have shown that inhibiting chemosensory systems leads to lifespan change in *C. elegans* and *D*. *melanogaster*. The first evidence regarding the effect of sensory systems on aging was revealed by Apfeld and Kenyon, who found that mutations that impair sensory neural structure and/or their function extend *C. elegans* lifespan (Apfeld and Kenyon, 1999). Various mutant worms with malformed sensory cilia, including *che-2*, *daf-10*, *daf-19*, and *osm-5* mutants, live up to 50% longer than wild-type worms. Lifespan is also increased by direct laser ablation of amphid sheath cells, which support the structure of amphid neurons (Apfeld and Kenyon, 1999). Alcedo and Kenyon used this technique to directly determine the roles of chemosensory neurons in *C. elegans* lifespan regulation (Alcedo and Kenyon, 2004). Laser ablation of either gustatory ASI and ASG neurons or olfactory AWA and AWC neurons prolongs lifespan. Gustatory and olfactory neurons appear to influence lifespan independently of each other because ablation of olfactory AWA and AWC neurons further lengthens the lifespan of gustatory ASI-ablated *C. elegans*. They also demonstrated that some normal chemosensory neurons limit *C. elegans* lifespan, while others appear to promote long lifespan. Laser ablation of either ASJ or ASK gustatory neurons does not influence lifespan in control worms and decreases the longevity resulting from ASI ablation, suggesting that ASJ and ASK neurons contribute to longevity in ASI-ablated animals (Alcedo and Kenyon, 2004). Overall, these pioneering studies established the role of *C. elegans* chemosensory neurons in the lifespan regulation at the organismal level.

Inhibiting components of chemosensory signal transduction extends lifespan. Mutations in *str-2*, which encodes a putative chemosensory GPCR, promote long lifespan (Alcedo and Kenyon, 2004). In addition, mutations in *kin-29*, a serine/threonine kinase that regulates the expression of a set of chemoreceptor genes, confer lifespan extension similar to that of other chemosensory mutants (Lanjuin and Sengupta, 2002). Multiple G proteins located downstream of chemosensory receptors in GPCR signaling cascades also influence longevity (Alcedo and Kenyon, 2004; Lans and Jansen, 2007; Hahm et al., 2009). Either loss-of-function mutations or overexpression of subsets of G proteins increases lifespan. Mutants in genes encoding Gα subunits including *gpa-1*, *gpa-5*, *gpa-9*, and *odr-3* live longer than wild type (Alcedo and Kenyon, 2004; Lans and Jansen, 2007; Hahm et al., 2009). Seemingly paradoxically, overexpression of *gpa-2*, *gpa-9*, or *gpa-11*, and constitutively active *gpa-3* mutations also lengthen lifespan (Lans and Jansen, 2007; Hahm et al., 2009). It seems likely that both decrease and increase in the activity of G proteins cause defects in chemosensation, which lead to life extension. Mutations in the cyclic nucleotide-gated channel subunit encoded by *tax-4* also extend lifespan at low temperatures (Apfeld and Kenyon, 1999; Lee and Kenyon, 2009). Together, these studies indicate that chemosensory signal transduction cascades regulate longevity in *C. elegans*.

Lifespan extension is observed in *C. elegans* mutants that are defective in neurosecretory processes. Mutations in *unc-31*, the *C. elegans* homolog of Ca^2+^-dependent activator protein for secretion (CAPS), or *unc-64*, the syntaxin required for synaptic transmission and neurosecretion, prolong lifespan (Ailion et al., 1999). In addition, mutations in *ocr-2*, which encodes a transient receptor potential vanilloid (TRPV) channel expressed in neurons, including chemosensory neurons, increase adult lifespan through the same genetic pathway as *unc-31* (Lee and Ashrafi, 2008). Interestingly, mutants that have defects in neurosecretion or sensory cilia display increased resistance to chronic starvation, as well as long lifespan. Increased survival of *unc-31* mutants during starvation is partially rescued by *unc-31* expression in ADL and ASH amphid chemosensory neurons, suggesting that perturbing neurosecretion in these chemosensory neurons underlies increased survival (Lee and Ashrafi, 2008). Together, these studies indicate that lifespan regulation by the chemosensory system is linked to neurosecretory system control.

Pharmacological perturbation of chemosensory neurons also increases lifespan. It has been shown that anticonvulsants used for treating seizure disorders in humans promote the longevity of invertebrate model animals; ethosuximide and trimethadione confer long lifespan in *C. elegans* (Evason et al., 2005; Collins et al., 2008) and lamotrigine extends lifespan in *D. melanogaster* (Avanesian et al., 2010). The Kornfeld group showed that treatment with ethosuximide lengthens *C. elegans* lifespan and prevents age-related physiological decline, such as decrease in feeding rates (Evason et al., 2005). Subsequently, Collins et al. screened for mutants resistant to high-dose ethosuximide-induced larval lethality and isolated those with defects in sensory cilium structure (Collins et al., 2008). They also showed that ethosuximide treatment abrogates chemotaxis in wild-type *C*. *elegans*, suggesting that the lifespan-increasing effects of ethosuximide act through inhibiting chemosensory function. The Buck group then performed a high-throughput chemical screen to identify anti-aging chemicals and discovered that mianserin and methiothepin prolong the lifespan of *C. elegans* (Petrascheck et al., 2007). These compounds are known to influence serotonin-mediated neural signaling and are used to treat depression in humans. Mianserin and methiothepin require serotonin receptor *ser-4* and a probable octopamine receptor *ser-3* to promote the longevity in worms (Petrascheck et al., 2007). Similar to the effects on the human serotonergic system, pre-incubation of mianserin or methiothepin antagonizes the actions of *C. elegans* SER-4 and SER-3 receptors in cultured mammalian cells, suggesting that these chemicals disrupt serotonin and octopamine neurotransmitter signaling to elicit lifespan-lengthening effects (Petrascheck et al., 2007).

The observations that chemosensory system impairment promotes longevity raise an important question of whether environmental chemical cues directly influence aging through sensory neurons. Worms fed with sub-strains of *E. coli* with different lipopolysaccharide (LPS) structures on the external cellular membrane have different lifespans (Soukas et al., 2009; Maier et al., 2010). Maier et al. raised the possibility that chemosensory neurons modulate the effects of consumed *E. coli* on lifespan probably through sensing the LPS structure (Maier et al., 2010). They further found that *nmur-1*, which encodes a homolog of neuromedin U receptor, mediates food type-dependent lifespan changes in *C. elegans* (Maier et al., 2010).

The role of the chemosensory system in lifespan regulation appears to be conserved in fruit flies. Pletcher's group demonstrated that the lifespan of flies is extended by mutations in the *Or83b* gene (Libert et al., 2007), which encodes an atypical co-receptor required for recruiting odorant receptors to ciliated dendrites of olfactory neurons (Larsson et al., 2004). In addition, nutrient-derived odorants directly accelerate aging in flies under dietary restriction, suggesting that dietary restriction increases lifespan at least partially through reducing the level of transmission of olfactory information to target cells and/or tissues (Libert et al., 2007). In addition to odorant sensation, a recent report suggests that carbon dioxide (CO_2_) sensation also regulates the aging of *D. melanogaster* (Poon et al., 2010). Mutations in the *Gr63a* gene, which encodes a CO_2_ olfactory receptor, or genetic ablation of CO_2_-sensing ab1C neurons increases the lifespan of *D. melanogaster* (Poon et al., 2010). Together, these findings strongly support the hypothesis that chemosensory systems use environmental cues to regulate lifespan in *D. melanogaster* and *C. elegans*.

### Signaling pathways that mediate the lifespan regulation by chemosensory systems

How does chemosensation influence lifespan? Although detailed mechanisms remain to be elucidated, recent studies suggest that chemosensory systems appear to affect lifespan at least partially by altering insulin/IGF-1 signaling in *C. elegans* (Figure 2). Activation of the insulin/IGF-1 receptor leads to the phosphorylation of downstream kinase cascades, including AGE-1/phosphoinositide-3 kinase (PI3K), phosphoinositide-dependent kinase 1 (PDK-1), AKT/protein kinase B (PKB), and serum- and glucocorticoid-induced kinase 1 (SGK-1) (Kenyon, 2010). This results in the subsequent phosphorylation of DAF-16/FOXO transcription factor, prevents its nuclear localization, and therefore inhibits its activation. When insulin/IGF-1 signaling is reduced, activated DAF-16/FOXO translocates to the nucleus and regulates the expression of downstream targets, including genes required for stress resistance, protein homeostasis, and lifespan extension (Kenyon, 2010).

Several lines of evidence support the idea that the *C. elegans* chemosensory system can influence lifespan through the insulin/IGF-1 signaling pathway. Mutations causing defects in sensory cilia, direct ablation of ASI chemosensory neurons, or defects in neurosecretion by *unc-64* or *unc-31* mutations do not further lengthen the lifespan of long-lived *daf-2*/insulin/IGF-1 receptor mutants, suggesting that chemosensory inhibition and reduced insulin/IGF-1 signaling act through the same genetic pathway (Ailion et al., 1999; Apfeld and Kenyon, 1999; Alcedo and Kenyon, 2004). In addition, inhibiting chemosensation in *C. elegans* appears to promote longevity by activating DAF-16/FOXO. Chemosensory defects caused by mutations in *daf-10*, *odr-3*, or *gpa-1* promote nuclear localization of DAF-16/FOXO (Lin et al., 2001; Lans and Jansen, 2007). Consistently, the expression level of *sod-3*, one of the direct targets of DAF-16/FOXO, is increased by chemosensory *daf-11* loss-of-function or *gpa-3* gain-of-function mutations (Hahm et al., 2009). Furthermore, longevity conferred by inhibiting chemosensory function or neurosecretion is largely dependent on DAF-16/FOXO (Ailion et al., 1999; Apfeld and Kenyon, 1999; Lanjuin and Sengupta, 2002; Alcedo and Kenyon, 2004; Lans and Jansen, 2007; Lee and Ashrafi, 2008). Together, these studies suggest that chemosensory perception influences the insulin/IGF-1 signaling pathway to regulate lifespan.

How do chemosensory defects in *C. elegans* modulate the insulin/IGF-1 signaling? A study by Ashrafi's laboratory showed that chemosensory neurons regulate insulin-like peptide (ILP) secretion (Lee and Ashrafi, 2008). They generated transgenic worms expressing red fluorescent protein (mCherry)-tagged *daf-28*, which encodes an ILP, under the control of an ADL sensory neuron-specific promoter. They found that *unc-31* or *ocr-2* mutations disrupt mCherry-DAF-28 release from ADL sensory neurons (Lee and Ashrafi, 2008), raising the possibility that loss of chemosensory function reduces the levels of ILPs, which may lead to longevity.

In addition to insulin/IGF-1 signaling, there seem to be other signaling pathways that influence lifespan downstream of chemosensory transduction, because the longevity of several chemosensory-defective animals is partially independent of DAF-16/FOXO. For example, mutations in *daf-10*, *osm-3*, *osm-5*, or *gpa-1* slightly but significantly increase *daf-16*/FOXO mutant lifespan (Apfeld and Kenyon, 1999; Lans and Jansen, 2007). Moreover, mutations in G protein genes such as *odr-3* and *gpa-11* further enhance the long lifespan of *daf-2*/insulin/IGF-1 mutants (Lans and Jansen, 2007). The identification of these insulin/IGF-1-independent components will be a crucial next step towards better understanding of how chemosensory systems influence lifespan.

### Chemosensation and mammalian aging

**Figure 3:**
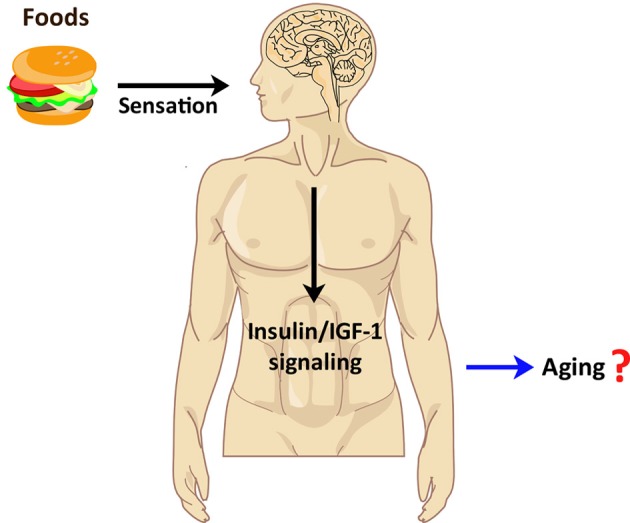
**Implication of chemosensory regulation in human aging.** Food perception by sensory systems in humans appears to increase human insulin/IGF-1 signaling. Because the insulin/IGF-1 signaling pathway is known to regulate mammalian lifespan, perhaps human chemosensation also influences aging via the insulin/IGF-1 pathway.

Although there is no evidence for a chemosensory neural system influencing the lifespan of mammals to date, it has been established that the mammalian nervous system controls endocrine signaling and influences lifespan. The regulation of aging by the insulin/IGF-1 signaling pathway is evolutionarily well conserved from yeast to mammals (Kenyon, 2010). Interestingly, mammalian insulin/IGF-1 signaling is modulated by a neural system as in *C. elegans*. Insulin signaling in the brain influences not only metabolism and reproduction but also lifespan in mice (Bruning, 2000; Plum et al., 2005; Taguchi et al., 2007; Kappeler et al., 2008; Scherer et al., 2011). Insulin levels in humans are increased by the sensation of foods (Sjostrom et al., 1980), suggesting that chemosensation itself can influence insulin signaling in mammals (Figure 3). It will be interesting to determine whether insulin signaling regulated by the mammalian chemosensory system influences aging processes.

## The role of thermosensory system in aging

### Thermosensory neural system

Sensing and responding to ambient temperature are vital processes for organisms to maintain their body functions. Environmental temperature fluctuations are tolerated by the homeostatic adjustment of body temperature by neuronal circuitry in warm-blooded animals (endotherms). In the case of cold-blooded animals (ectotherms), body temperature cannot be adjusted and is dependent on ambient temperature; therefore, locomotion towards the optimum temperature is the most suitable strategy for survival (Garrity et al., 2010). Thus, environmental temperature can have drastic effects on ectotherm physiology, especially in small organisms. In principle, thermoregulatory behaviors are triggered by thermosensory receptors located on the surface or inside of the body (Garrity et al., 2010).

Along with stimulants such as mechanical forces and chemical cues, temperature is perceived by sensory neurons in the peripheral nervous system, which originate from dorsal root ganglia (DRG) and trigeminal ganglia (TG) in mammals. These neurons have two nerve endings with a branched axonal structure. One nerve ending innervates peripheral tissues and contains a variety of receptors to perceive stimuli, including temperature, and the other connects to the spinal cord to transmit these stimuli (Dhaka et al., 2006). Temperature-sensitive neurons localized in the preoptic area (POA) and anterior hypothalamus are the major regulators of mammalian body temperature homeostasis (Morrison and Nakamura, 2011). The POA is thought to limit core body temperature fluctuations regardless of extreme environmental temperature changes (Clapham, 2011). Slight temperature changes perceived by the POA or lateral hypothalamus are almost immediately balanced by body temperature regulation (Clapham, 2011). At the molecular level, the transient receptor potential (TRP) family of cation channels is thought to be the major mammalian thermosensor (Morrison and Nakamura, 2011). TRPM8 and TRPA1 are cold-sensitive channels, whereas four TRPV channels (TRPV1-4) are activated by warm temperatures (Morrison et al., 2008; Morrison and Nakamura, 2011).

*C. elegans* and *D. melanogaster* have served as useful model animals to characterize thermosensory perception and thermo-regulatory pathways. The *C. elegans* thermosensory system allows movement towards temperatures previously associated with the existence of food and movement away from those temperatures associated with food deprivation. It has been shown that AFD, AWC, and ASI neurons respond to temperature changes, and play key roles in these temperature-associated thermotactic behaviors (Mori and Ohshima, 1995; Biron et al., 2008; Kuhara et al., 2008; Beverly et al., 2011). Among them AFD neurons are the major thermosensory neurons, which form neural circuits with AIY, AIZ, and RIA interneurons (Mori and Ohshima, 1995; Mori et al., 2007). Temperature shifts are perceived by AFD thermosensory neurons upon exceeding the threshold temperature, which is likely stored as a memory acquired during previous growth conditions. At the molecular level, AFD neurons are activated by cGMP-dependent cation channels composed of TAX-2 and TAX-4 subunits, the functions of which rely on cGMP production by AFD thermosensory neuron-specific guanylyl cyclases GCY-8, GCY-18, and GCY-23 (Inada et al., 2006). Increased ambient temperature appears to result in the activation of these GCYs, which promotes the elevation of intracellular cGMP levels (Wasserman et al., 2011). This leads to TAX-2/TAX-4 cation channel opening, which causes cation flux into AFD neurons (Satterlee et al., 2004).

Unlike worms, the temperature preference of fruit flies is mostly constant within a small range, rather than experience dependent. *Drosophila* first instar larvae exhibit negative thermotaxis above 30°C and positive thermotaxis below 20°C towards their preferred temperatures, which range between 24–28°C (Garrity et al., 2010). Although it is known that the temperature preference of adult flies is similar to that of larvae, their thermotactic behaviors and the involvement of thermosensation are not well understood due to the free movement of adult flies (Garrity et al., 2010). The sensation of cool or warm temperatures requires three types of thermosensory TRP channels: dTRPA1, Pyrexia (dTRPA2), and Painless (Garrity et al., 2010). dTRPA1 is activated at warm temperatures with a threshold of 27°C in a heterologous system (Viswanath et al., 2003) and is important for larval thermotaxis (Rosenzweig, 2005). Pyrexia is activated by temperatures over 40°C *in vitro*, and *pyrexia* mutants display defects in thermotolerance (Lee et al., 2005). Painless is activated at temperatures higher than 42°C *in vitro* (Sokabe et al., 2008) and helps flies avoid noxious heat (McKemy, 2007).

### Regulation of longevity in *C. elegans* by the thermosensory system

**Figure 4:**
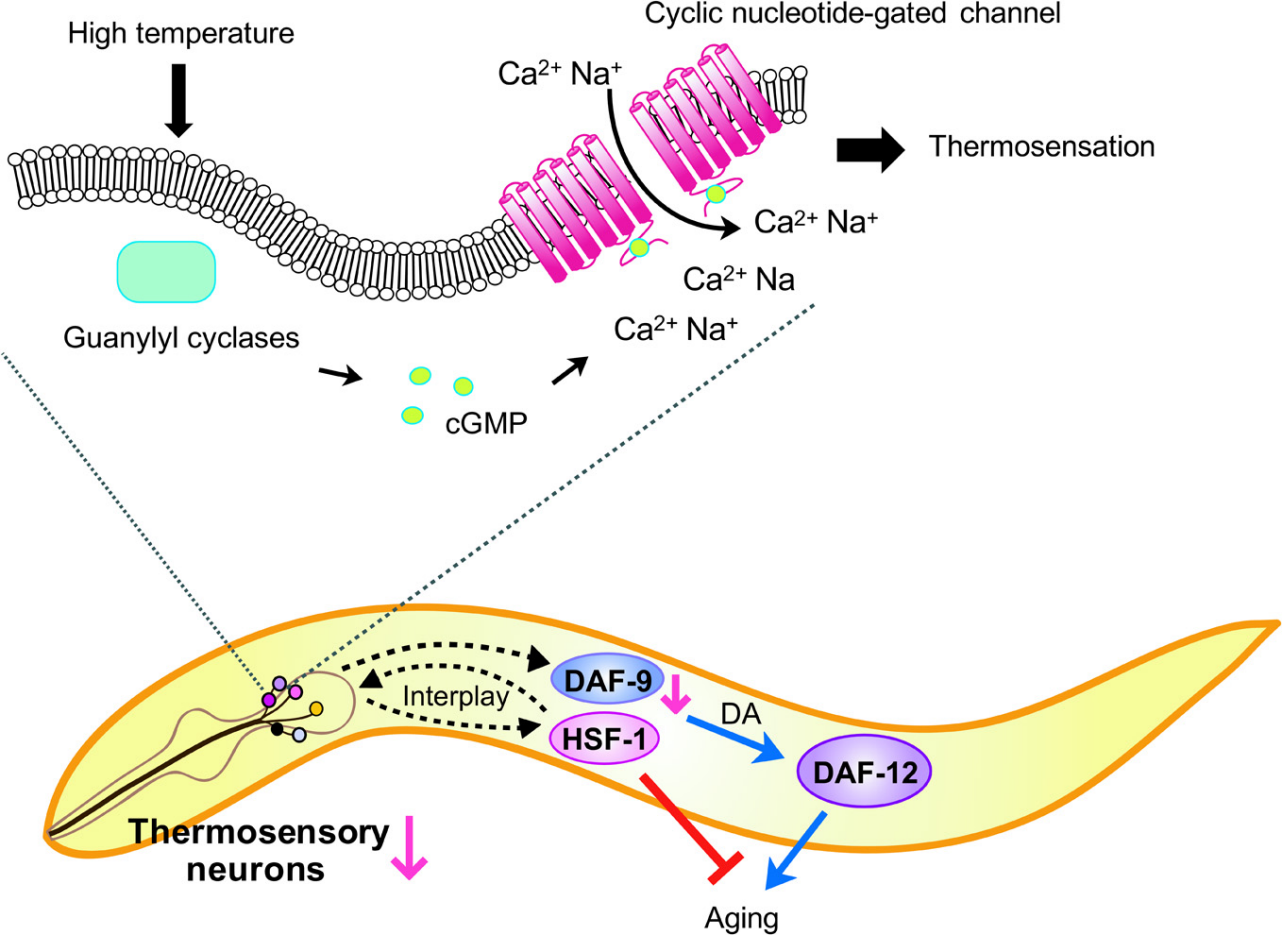
**Model of *C. elegans* lifespan control by thermosensory AFD neurons.** Increased ambient temperature is perceived by AFD thermosensory neurons. Although the exact mechanism for thermal cue perception is not understood, upon sensing temperature elevation, G protein signaling promotes cyclic GMP production and facilitates the influx of Ca^2+^ and Na^+^ cations by opening cyclic nucleotide-gated channels (CNGs). The signal perceived by the thermosensory neurons is transmitted to downstream effectors, such as DAF-12/NHR and heat shock factor 1 (HSF-1), via intercellular (cell-nonautonomous) signaling. Impairment of AFD thermosensory neurons reduces DAF-9/CYP levels, which in turn decreases the level of sterol hormones, dafachronic acids (DA). This leads to altered DAF-12/NHR activity, which accelerates aging at high temperature. In addition, AFD neurons and HSF-1 regulate each other in a cell-nonautonomous manner to affect physiological processes of whole worms. Defects in thermosensory neurons decrease the activity of HSF-1 in non-neuronal tissues, while impairment of *hsf-1* in non-neuronal tissues decreases thermosensory neural function. It has also been shown that inhibiting HSF-1 speeds up aging.

Despite relatively well-defined thermosensory system structures and functions for the perception of ambient temperature, the effects of these systems on the regulation of animal physiology, including aging, remain poorly understood. Lee and Kenyon demonstrated that AFD thermosensory neurons regulate worm lifespan through a sterol endocrine signaling pathway at high temperatures (Lee and Kenyon, 2009) (Figure 4). Impairment of AFD neurons by either laser ablation or mutations causes a significant lifespan decrease at high temperature (25°C) but have no effect at low temperatures (20 and 15°C). Perturbation of AFD neurons reduces the expression of *daf-9*, which encodes a cytochrome P450 (CYP) that is responsible for producing the sterol hormones known as dafachronic acids. This subsequently influences the activity of DAF-12, a nuclear hormone receptor (NHR) whose activity is regulated by dafachronic acids. They proposed a model in which AFD neurons stimulate *daf-9*/*CYP* expression, which in turn regulates DAF-12/NHR activity and leads to altered lifespan at high temperature. Another noteworthy study suggested that AFD thermosensory neurons regulate the transient heat shock response of whole worms upon perception of acute high temperature (Prahlad et al., 2008). When mutant worms that have defects in AFD thermosensory neurons are exposed to transient heat shock, the expression of heat-shock responsive chaperone genes in neurons and other tissues are reduced. In addition, these animals are less tolerant to heat shock than wild-type worms. A subsequent study showed that regulation of chaperone gene expression by AFD neurons also influences protein aggregation in a neurodegenerative disease model (Prahlad and Morimoto, 2011). Therefore, signaling from thermosensory neurons to other tissues appears to mediate the proper heat shock response of whole worms. The induction of heat-shock responsive genes by AFD neurons is solely dependent on the heat shock factor-1 (HSF-1), which is a leucine-zipper transcription factor crucial for various biological functions, including heat-shock response, lifespan regulation, and organismal development (Neef et al., 2011). The interplay between the thermosensory neural system and HSF-1 was further demonstrated by Sugi et al. who found that HSF-1 is required for the thermotactic behavior of worms towards a preferred cultivation temperature (Sugi et al., 2011). Non-neuronal expression of *hsf-1* is sufficient to rescue thermotactic defects and does so by regulating AFD neurons through estrogen signaling. Thus, regulation of HSF-1 activity in non-neuronal cells, as well as AFD neurons in *C. elegans* appears to act as thermosensors. Interestingly, both AFD thermosensory neurons and the HSF-1 transcription factor are required for maintaining normal worm lifespan at high temperatures (Lee and Kenyon, 2009). It is tempting to speculate that AFD neuronal signaling and HSF-1 activity regulation in the non-neuronal cells of ectothermic animals, such as *C. elegans*, operate together to properly tune physiological processes, including aging (Figure 4).

### Implications in mammalian aging

Although there is no known relationship between the thermosensory system and mammalian aging, there are hypotheses that mammalian thermosensory and thermoregulatory systems may influence aging, as well as other biological processes. Hibernation is defined as the controlled and reversible reduction and/or inhibition of body temperature, metabolic rate, oxygen consumption, and other physiological activities (Geiser, 2004). An early study on the relationship between thermosensation and hibernation showed that reduced body temperature and arousal from deep hibernation are sensed and regulated by the POA of the hypothalamus in hibernating mammals (Heller and Hammel, 1972). The role of hibernation in mammalian longevity has long been debated, and direct data have been scarce to date. Most bats hibernate during the winter and are known to have long lifespan compared to mammals of similar size and metabolic rate; bats can live as long as 30 years, whereas the maximum lifespans of mice and rats are 4 and 5 years, respectively (Brunet-Rossinni and Austad, 2004). Wilkinson and South found that hibernating bats live approximately 6 years longer than non-hibernating bats on average (Wilkinson and South, 2002). Lyman et al. suggested that hibernation also prolongs the lifespan of the Turkish hamster *Mesocricetus brandti* (Lyman et al., 1981). Animals that hibernate for a longer time (19–33% of their lives) live ~50% longer than animals that hibernate for a shorter time (0–11% of their lives) at cold temperatures. It has been proposed that hibernators keep their metabolic rates at very low levels, which may lead to an extended lifespan, while animals that are exposed to cold and do not take the advantage of hibernation may suffer from cold stress (Lyman et al., 1981).

**Figure 5:**
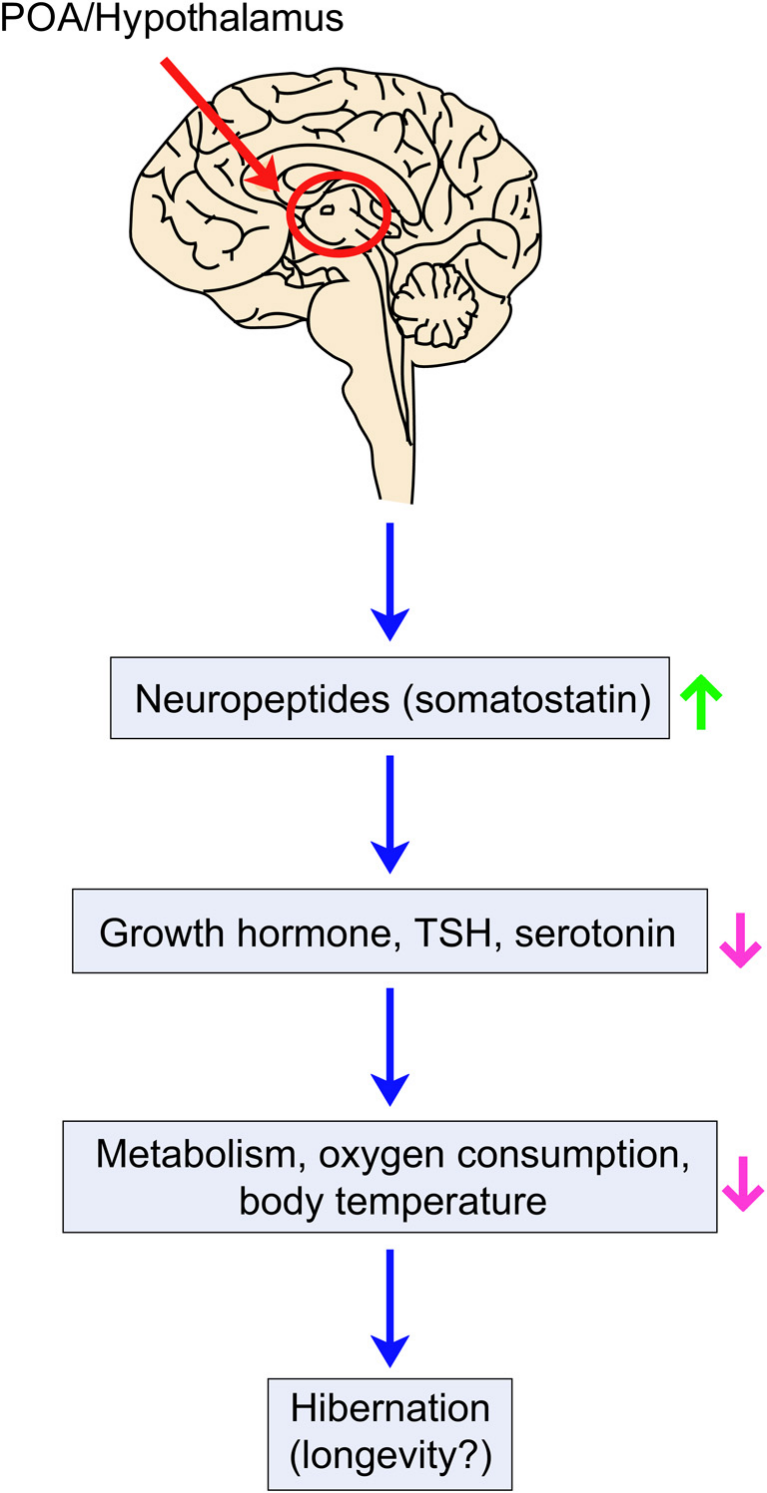
**Hypothetical model of longevity and hibernation modulation by thermoregulation in mammals.** Ambient temperature decrease is sensed by cold-activated receptors and transmitted to the POA of the hypothalamus. Neuropeptides such as somatostatin may be secreted to inhibit growth, cellular proliferation, body core temperature, oxygen consumption, and metabolic activities by decreasing the levels of growth hormone, thyroid-stimulating hormone (TSH) and/or serotonin. These changes appear to contribute to the hibernation process and may influence longevity.

What are the potential molecular mechanisms behind the beneficial effects of hibernation on health and longevity? Recent research using the Djungarian hamster *Phodopus sungorus* suggests that changes in relative telomere length (RTL) may underlie the potential benefits of hibernation (Turbill et al., 2011). Djungarian hamsters use daily torpor, which can be considered as temporary hibernation, upon exposure to winter conditions for over 180 days. Animals that are kept at cold temperature (9°C) and use daily torpor have increased RTL compared to animals kept at warm temperature (20°C). Therefore, the use of daily torpor may delay aging during harsh environmental conditions by increasing telomere length in hamsters. Other studies suggest that endocrine signaling plays a role in hibernation and perhaps the longevity associated with it. The level of neuropeptide somatostatin, a negative regulator of growth hormone and thyroid-stimulating hormone (TSH) (Tichomirowa et al., 2005), increases before hibernation in the golden-mantled squirrel *Spermaphilus lateralis* (Muchlinski et al., 1983). Interestingly, growth hormone knock-out mice have long lifespan (Coschigano et al., 2000), significantly reduced levels of thyroid hormone, and decreased body core temperature compared to normal mice (Hauck et al., 2001). In addition, Ames dwarf mice, which are deficient in growth hormone, TSH, and prolactin, are long lived (Brown-Borg et al., 1996). A recent study suggests that growth hormone receptor deficiency in humans is associated with reduced incidences of age-related diseases, including cancer and diabetes (Guevara-Aguirre et al., 2011). Collectively, one can speculate that before the onset of hibernation, somatostatin inhibits growth hormone and TSH secretion, leading to lifespan extension (Figure 5).

One of the common features of mammalian hibernation is decreased body temperature. Is there a cause-and-effect relationship between body temperature and aging? One striking report showed that reducing core body temperature significantly prolongs lifespan (Conti et al., 2006). The method was based on an assumption that temperature increase in the lateral hypothalamus, which is in close proximity to thermosensory neuron-rich POA, would lead to a counter-balanced response to decrease core body temperature. Overexpression of uncoupling protein 2 (UCP2), which generates heat by uncoupling the mitochondrial proton gradient and oxidative phosphorylation, in hypocretin neurons in mice elevates the level of heat perceived by thermosensory neurons located in the POA and lateral hypothalamus. This leads to a decrease in core body temperature and a subsequent reduction of energy requirement. These long-lived transgenic mice show diminished levels of free radicals and oxidative stress. Their prolonged lifespan may be due to similar mechanisms in dietary restriction-induced longevity, as the reduced core body temperature leads to more efficient energy consumption and lowered toxic metabolic byproduct production, which are similarly observed upon dietary restriction (Conti et al., 2006). It will be interesting to test whether thermosensory neurons affect lifespan extension induced by decreased body temperature.

## Concluding remarks

In this review, we discussed studies regarding the effects of sensory perception on aging. Although over a decade of research have established a role for sensory systems in the lifespan regulation of *C. elegans* and *D. melanogaster*, many important questions remain unanswered. It will be interesting to test whether insulin/IGF-1 signaling mediates the lifespan effects of chemosensory neurons and whether thermosensory neurons affect the aging processes in *D. melanogaster*. Also, it will be crucial to identify genes and signaling pathways, other than the insulin/IGF-1 pathway, which are involved in lifespan modulation by chemosensation. Perhaps the most important question in the field is whether mammalian sensory systems influence aging. Because many findings on aging regulation in invertebrate model organisms have been shown to be conserved in mammals, including humans, it would not be surprising to find homologous mechanisms in mammals. If so, it will be a fascinating starting point for future research, including anti-aging drug screens, because mammalian sensory systems are relatively well understood at the molecular level; therefore, components in sensory systems, such as receptors, can be modulated to promote healthy aging.

### Conflict of interest statement

The authors declare that the research was conducted in the absence of any commercial or financial relationships that could be construed as a potential conflict of interest.
